# PoCos: Population Covering Locus Sets for Risk Assessment in Complex Diseases

**DOI:** 10.1371/journal.pcbi.1005195

**Published:** 2016-11-11

**Authors:** Marzieh Ayati, Mehmet Koyutürk

**Affiliations:** 1 Electrical Engineering and Computer Science Department, Case Western Reserve University, Cleveland, Ohio, United States of America; 2 Center of Proteomics and Bioinformatics, Case Western Reserve University, Cleveland, Ohio, United States of America; University of Pennsylvania, UNITED STATES

## Abstract

Susceptibility loci identified by GWAS generally account for a limited fraction of heritability. Predictive models based on identified loci also have modest success in risk assessment and therefore are of limited practical use. Many methods have been developed to overcome these limitations by incorporating prior biological knowledge. However, most of the information utilized by these methods is at the level of genes, limiting analyses to variants that are in or proximate to coding regions. We propose a new method that integrates protein protein interaction (PPI) as well as expression quantitative trait loci (eQTL) data to identify sets of functionally related loci that are collectively associated with a trait of interest. We call such sets of loci “population covering locus sets” (PoCos). The contributions of the proposed approach are three-fold: 1) We consider all possible genotype models for each locus, thereby enabling identification of combinatorial relationships between multiple loci. 2) We develop a framework for the integration of PPI and eQTL into a heterogenous network model, enabling efficient identification of functionally related variants that are associated with the disease. 3) We develop a novel method to integrate the genotypes of multiple loci in a PoCo into a representative genotype to be used in risk assessment. We test the proposed framework in the context of risk assessment for seven complex diseases, type 1 diabetes (T1D), type 2 diabetes (T2D), psoriasis (PS), bipolar disorder (BD), coronary artery disease (CAD), hypertension (HT), and multiple sclerosis (MS). Our results show that the proposed method significantly outperforms individual variant based risk assessment models as well as the state-of-the-art polygenic score. We also show that incorporation of eQTL data improves the performance of identified POCOs in risk assessment. We also assess the biological relevance of PoCos for three diseases that have similar biological mechanisms and identify novel candidate genes. The resulting software is publicly available at http://compbio.case.edu/pocos/.

## Author Summary

Several studies try to predict the individual disease risk using genetic data obtained from genome wide association studies (GWAS). Earlier studies only focus on individual genetic variants. However, studies on disease mechanisms suggest the aggregation of genomic variants may contribute to diseases. For this reason, researchers commonly use prior biological knowledge to identify genetic variants that are functionally related. However, these approaches are often limited to variants that are in the coding regions of genes. However, several risk variants are in the regulatory region. Here, we incorporate known regulatory and functional interactions to find sets of genetic variants which are informative features for risk assessment. Our result on seven complex diseases show that our method outperforms individual variant based risk assessment models, as well as other methods that integrate multiple genetic variants.

## Introduction

Genome-wide association studies (GWAS) have a transformative effect on the search for genetic variants that are associated with complex traits, since they enable screening of hundreds of thousands of genomic variants for their association with traits of interest [1]. Recently published GWAS lead to the discovery of susceptibility loci for many complex diseases, including type 2 diabetes [2], psoriasis [3], multiple sclerosis [4], and prostate cancer [5]. For improved identification of risk variants, researchers draw information from clinical, microarray, copy number, and single nucleotide polymorphism (SNP) data to build disease risk models, which are then used to predict an individual’s susceptibility to the disease of interest [6, 7]. Several companies, such as deCODE genetics (http://www.decode.com) and 23andme (https://www.23andme.com) have started using SNPs identified by GWAS, to provide personal genomic test services in the United States and health related genomic test services in Canada and the United Kingdom.

An important problem with GWAS is that the identified variants account for little heritability [8, 9]. However, empirical evidence from model organisms [10] and human studies [11] suggests that the interplay among multiple genetic variants contribute to complex traits. Epistasis among pairs of loci, i.e., significantly improved association with the phenotype when two loci are considered together, is also shown to provide provide further insights into disease mechanisms [12–14]. Therefore, recent studies focus on identifying the interactions among pairs of genomic loci, as well as among multiple genomic loci [15–17]. These studies suggest that consideration of more than one locus together can better capture the relationship between genotype and phenotype. For this reason, genetic markers that aggregate multiple genomic loci can be used to design effective strategies for risk assessment and guide treatment decisions [18].

The Polygenic score is a commonly used method to identify the joint association of a large mass of the loci to predict disease risk [19]. The first application of polygenic score on GWAS data shows that the genetic risk for schizophrenia is a predictor of bipolar disorder [20]. There are also several studies demonstrating that polygenic risk score is a powerful tool in risk prediction [20–22]. However, polygenic score does not make use of prior biological knowledge, which may be useful in generating more robust features by incorporating the functional relationships among individual variants. Furthermore, according to a recent comparative assessment of various classification algorithms, there are no statistically significant differences between state-of-the-art classification algorithms in terms of performance in risk assessment [23]. This observation suggests that research on construction of features for risk assessment can be useful in improving the classification performance of these algorithms.

Since detection of epistasis and higher order interactions is computationally expensive, many methods first assess the disease association of individual loci and then use functional knowledge to integrate these associations [24–26]. The key idea behind these methods is that functionally related variants, e.g., those that induce dense subnetworks in protein-protein interaction (PPI) networks, can provide stronger statistical signals when they are considered together [27]. Based on similar insights, some researchers integrate GWAS with pathway information to identify statistically significant pathways that are associated with the disease [28, 29]. Recently, Azencott *et al.* propose a method to discover sets of genomic loci that are associated with a phenotype while being connected in an underlying biological network [30]. They use an additive model to integrate the genotypes of loci and use connectivity patterns in the network to select a functionally coherent set of disease associated SNPs. While this method works on a network of genomic loci, the network is constructed based on the interactions among genes and mapping of loci to genes. For this reason, the application of these methods is limited to the variants in coding regions or in regions that are in close proximity to genes. However, 88 percent of genotyped variants in GWAS fall outside of coding regions [31]. Several risk variants are found in non-coding regions of the genome and it is shown that the functional effects of these variants are regulatory (e.g., mRNA expression, microRNA expression) as opposed to directly influencing protein structure or function [32].

In this paper, we propose a new algorithm for the identification of multiple functionally related genomic variants that are collectively associated with a phenotype. The proposed method builds on the concept of “Population Covering Locus Sets” (PoCos) [33, 34]. A PoCo is a set of loci that harbor at least one susceptibility allele in samples with the phenotype of interest. Here, we extend the notion of PoCos to enable adaptive identification of “susceptibility genotype” (as opposed to susceptibility allele) for each locus. We also develop a method for aggregating the genotypes of multiple loci in a PoCo to compute representative genotypes for use in risk assessment. Finally, in order to capture the functional relationship between genomic loci, we integrate GWAS data with human protein-protein interaction (PPI) network and regulatory interactions identified via expression quantitative trait loci (eQTL).

We use the PoCos identified by the proposed framework to construct features that can be used in risk assessment. We evaluate the performance of PoCos in risk assessment via cross-validation on seven GWAS case-control data sets obtained from the Wellcome Trust Case-Control Consortium (WTCCC). We compare the risk assessment performance of models built using PoCos to that of models built using individual loci and polygenic score. Our experimental results show that PoCos significantly outperform individual loci and polygenic score in risk assessment. Furthermore, we assess the information added by the incorporation of PPI and eQTL and observe that inclusion of these data leads to more parsimonious models for risk assessment.

In the next section, we describe the proposed procedure for modeling the genotypes and identifying PoCos. Then we describe how we use PoCos to develop a model for risk assessment. Subsequently, we present comprehensive experimental results on GWAS data sets for Type 2 Diabetes (T2D), Psoriasis (PS), Type 1 Diabetes (T1D), Hypertension (HT), Bipolar Disorder (BD), Multiple Sclerosis (MS) and Coronary Artery Disease (CAD). Our results show that the proposed method significantly outperforms individual variant based risk assessment model as well as the state-of-the-art polygenic score. We also observe that integrating prior biological information leads to more parsimonious models for risk assessment.

## Methods

**Fig 1:**
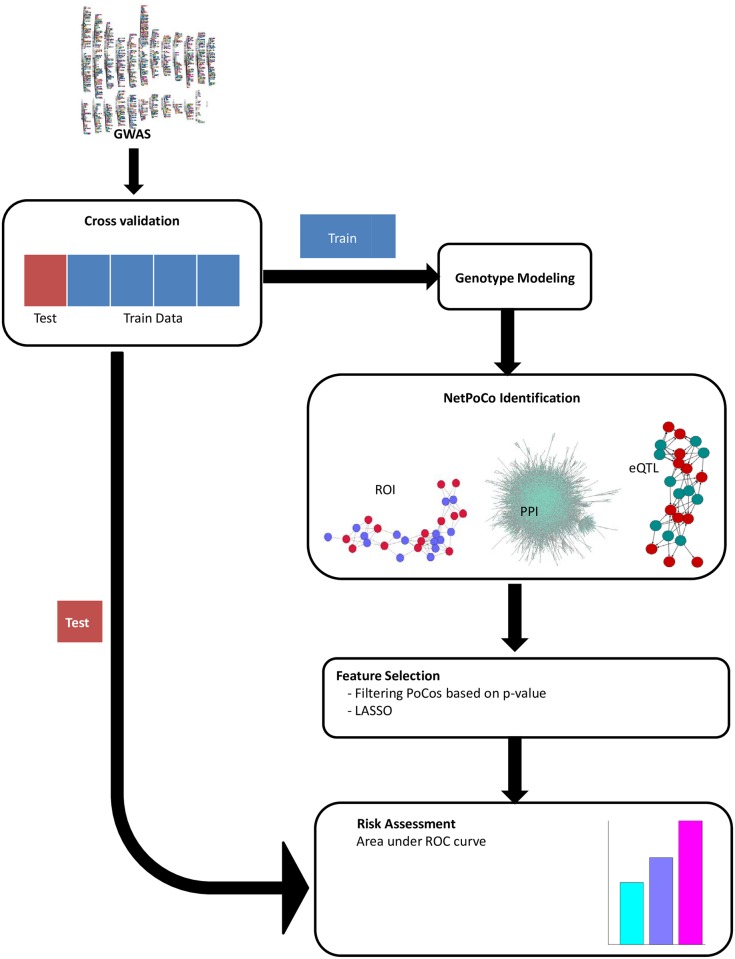
The workflow of the proposed method for the identification of PoCos and their utilization in risk assessment.

In this section, we first present the set-up for genome-wide association studies. We then define “Population Covering Locus Sets” (PoCos) and describe the algorithm we use to identify PoCos. Finally, we describe our feature selection framework for the selection of PoCos to be used for risk assessment. The workflow of the proposed method is presented in Fig 1.

### Genome-Wide Association Data

The input to the problem is a genome-wide association (GWA) dataset *D* = (*C*, *S*, *g*, *f*), where *C* denotes the set of genomic loci that harbor the genetic variants (e.g., single nucleotide polymorphisms or copy number variants) that are assayed, *S* denotes the set of samples, *g*(*c*, *s*) denotes the genotype of locus *c* ∈ *C* in sample *s* ∈ *S*, and *f*(*s*) denotes the phenotype of sample *s* ∈ *S*. Here, we assume that the phenotype variable is dichotomous, i.e., *f*(*s*) can take only two values: if sample *s* is associated with the phenotype of interest (e.g. diagnosed with the disease, responds to a certain drug etc.), *s* is called a “case” sample (*f*(*s*) = 1), otherwise (e.g., was not diagnosed with the disease, does not respond to a certain drug etc.), *s* is called a “control” sample (*f*(*s*) = 0). We denote the set of case samples with *S*_1_ and the set of control samples with *S*_0_, where *S*_1_ ∪ *S*_0_ = *S*. While we focus on qualitative traits here for brevity, the proposed methodology can also be extended to quantitative traits (i.e., when *f*(*s*) is a continuous phenotype variable).

### Identifying Genotypes of Interest

The minor allele for a locus is usually defined as the allele that is less frequent in the population. While it is common to focus on the minor allele as the risk allele, specific genotypes can also be associated with a phenotype [35–37]. Different types of encoding may represent different biological assumptions. In an additive model, each genotype is encoded as a single numeric feature that reflects the number of minor alleles (homozygous major, heterozygous, and homozygous minor are respectively encoded as 0, 1 and 2). This model does not capture combinatorial relationships between locus genotypes and phenotype, since the assumption is that one of the alleles quantitatively contributes to risk. In the recessive/dominant model, each genotype is encoded as two binary features (presence of minor allele and presence of major allele). This model does not capture the difference between homozygous and heterozygous genotypes, since it only accounts for the presence of an allele. Here, we argue that considering the effect of all possible genotype combinations can provide more information in distinguishing case samples from control samples. The five models proposed here capture all potential relationships, in that differences in heterozygosity vs. homozygosity, presence vs. absence of a specific risk allele are represented by different genotype models. This notion is particularly useful when the genotypes of multiple loci are being integrated. For example, heterozygosity on one locus can be associated with increased susceptibility to a disease, while homozygous minor allele on another locus may be protective at the presence of heterozygosity in the former locus [38]. In this case, the interaction between the two loci can be detected by considering the association of all possible genotype combinations with the phenotype.

We adaptively binarize the genotypes of each locus by considering all possible allele combinations. Given the genotype of a locus, we consider five different binary genotype models *m*^(*i*)^, *i* ∈ {1, … 5}. Based on each model, we generate a binary genotype profile for each locus. Namely, we consider the following genotype models:

**1. Homozygous Minor Allele:** This corresponds to the case when the possible effect of the minor allele is “recessive”, i.e., the locus is considered to harbor a genotype of interest if both copies contain the minor allele.

m(1)(c,s)=12ifg(c,s)∈{aa}0otherwise (1)

**2. Heterozygous Genotype:** The locus is considered to harbor a genotype of interest if the two copies contain different alleles.

m(2)(c,s)=12ifg(c,s)∈{Aa}0otherwise (2)

**3. Homozygous Major Allele:** The locus is considered to harbor a genotype of interest if both copies contain the major allele.

m(3)(c,s)=12ifg(c,s)∈{AA}0otherwise (3)

**4. Presence of Minor Allele:** This corresponds to the case when the possible effect of the minor allele is “dominant”, i.e., the locus is considered to harbor a genotype of interest if at least one copy contains the minor allele. This is the complement of *m*^(3)^.

m(4)(c,s)=12ifg(c,s)∈{Aa,aa}0otherwise (4)

**5. Presence of Major Allele:** The locus is considered to harbor a genotype of interest if at least one copy contains the major allele. This is the complement of *m*^(1)^.

m(5)(c,s)=12ifg(c,s)∈{Aa,AA}0otherwise (5)

Note that, although models *m*_4_ and *m*_5_ are complements of other models, we consider them separately. This is because, as we discuss in the next section, the 1s and 0s in the binary genotype profiles are considered asymmetrically while integrating the genotypes of multiple loci. Also note that “homozygous minor allele or homozygous major allele” is not considered since it is not associated with a specific risk allele.

**Fig 2:**
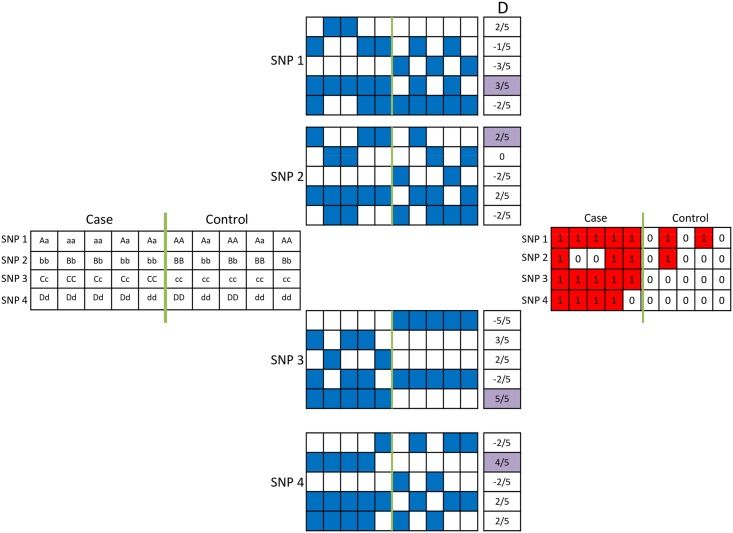
Model selection and computation of binary genotype profiles for each genomic locus. The genotypes of four loci on a hypothetical case-control dataset are shown on the left. The five possible binary genotype profiles for each locus are computed, as shown in the middle. Blue squares indicate the presence of the genotype of interest in the respective sample for each model (respectively, homozygous minor allele, heterozygous, homozygous major allele, presence of minor allele, presence of major allele). The resulting binary genotype profiles for each locus are shown on the right. Red squares indicate the existence of genotype of interest according to the selected model. In this example, models *m*^(4)^, *m*^(1)^, *m*^(5)^, and *m*^(2)^ are respectively selected for the four loci.

To select a genotype model for each locus, we separately assess the association of the resulting five genotype profiles with the phenotype of interest. Subsequently, we choose the model that leads to greatest discrimination between cases and controls, and use the respective binary genotype profile as the representative genotype of that locus. This process is illustrated in Fig 2.

For each locus *c*, binarization according to the five different genotype models produces five |*S*|-dimensional binary genotype profiles *m*^(*i*)^(*c*), *i* ∈ {1, … 5}. For each binary genotype profile *m*^(*i*)^(*c*), we compute the difference in the fraction of case and control samples that harbor the genotype of interest as follows:

$$D^{\left(i\right)}\left(c\right)=\frac{\langle f,m^{\left(i\right)}\left(c\right)\rangle }{|S_{1}|}-\frac{\langle 1-f,m^{\left(i\right)}\left(c\right)\rangle }{|S_{0}|}.$$ (6)

where **1** denotes a vector of all 1’s and <.> denotes the inner product of two vectors. We then determine the binary genotype model for each locus as the model that maximizes the difference of relative coverage between case samples and control samples, i.e.:

$$k\left(c\right)={\text{argmax}}_{i\in \{1\cdots 5\}}\left\{\right|D^{\left(i\right)}\left(c\right)\left|\right\}.$$ (7)

Based on the selected model for each locus, we compute the binary genotype profile accordingly:

$$M\left(c,s\right)=m^{\left(k(c)\right)}\left(c,s\right).$$ (8)

### Population Covering Locus Sets (PoCos)

Once we compute the binary genotype profiles for all loci, we identify Population Covering Locus Sets (PoCos). In previous work, we define and use PoCos in the context of prioritizing locus pairs for testing epistasis [33]. In this earlier definition, the genotypes of interest are limited to the presence of the minor or major allele; i.e., only the last two models described in the previous section are used to determine the binary genotype profile of each locus. Here, we generalize the concept of PoCo to utilize five different models for determining the genotypes of interest, as described in the previous subsection.

A PoCo is a set of genomic loci that collectively “cover” a larger fraction of case samples while minimally covering control samples. Namely for a given set *P* ⊆ *C* of loci, we define the set of case and control samples covered by *P* respectively as

$$E\left(P\right)=\cup_{c\in P}\left\{s\in S_{1}:M\left(c,s\right)=1\right\}$$ (9)

and

$$T\left(P\right)=\cup_{c\in P}\left\{s\in S_{0}:M\left(c,s\right)=1\right\}.$$ (10)

We define a PoCo as a set *P* of loci that satisfies |*E*(*P*)| = |*S*_1_| while minimizing |*T*(*P*)|. Note that, since we are interested in finding all sets of loci with potential relationship in their association with phenotype, we do not define an optimization problem that aims to find a single PoCo with minimum |*T*(*P*)|. We rather develop an algorithm to heuristically identify all non-overlapping PoCos with minimal |*T*(*P*)|.

### Identification of PoCos

To identify all non-overlapping PoCos, we use a greedy algorithm that progressively grows a set of loci to maximize the difference of the fraction of case and control samples covered by the loci that are recruited in a PoCo. In another words, we initialize *P* to ∅ and at each step, add to *P* the locus that maximizes

$$\delta \left(c\right)=\left|\frac{E\left(\left\{c\right\}\right)\cap S^{\prime }|}{|S_{1}|}-\frac{|T\left(\left\{c\right\}\right)\cap S^{\prime }|}{|S_{0}|}\right|$$ (11)

where *S*′ = *S*\(*E*(*P*) ∪ *T*(*P*)). The algorithm stops when all case samples are covered. We then record *P*, remove the loci in *P* from the dataset, and identify another PoCo. This process continues until it is not possible to find a set of loci that covers all case samples.

We develop two methods to identify two different types of PoCos. The first type of PoCos (named “network-free PoCos”) are identifed using the greedy algorithm described above, without the use of any prior biological information. The second type of PoCos are NetPocos, which are identified by restricting the search space to connected subgraphs of a network of potential functional relationships among genomic loci. As we describe below, this network is constructed by integrating established locus-gene associations from eQTL studies and protein-protein interaction (PPI) data that contains functional relationships among genes.

#### Network-free PoCos

For network-free PoCos, the search space for the problem contains all the loci that are genotyped and no restriction is applied on the search space. We use *δ*(.) to guide the search for PoCos, and require the search to proceed until all case samples are covered.

#### NetPocos

Since our aim is to find sets of variants that are related to each other in their association with a phenotype, interaction data can provide a useful functional context for PoCos. This approach is inspired by the NetCover algorithm that is used to identify dysregulated subnetworks in the context of cancer [39]. To identify NetPocos, in addition to GWAS data, we utilize a *heterogeneous network* *G* = (*V* ∪ *U*, *E* ∪ *F* ∪ *Q*) that represents the functional relationships among genomic loci. The network contains two types of nodes: genomic loci and genes/proteins. More precisely, the set *U* ⊆ *C* contains all genomic loci that are genotyped in the GWAS and are located in the gene region of interest or are expression quantitative trait loci. The set *V* contains all human genes/proteins.

The interactions and associations between these nodes are represented by three different sets of edges:

- *F* contains an edge between locus *c* ∈ *U* and gene *v* ∈ *V* if *c* is in the region of interest (RoI; defined as 50Kb up- and down-stream of the coding region in our experiments) of *v*. We call these edges *RoI edges*.
- *Q* contains an edge between locus *c* ∈ *U* and gene *v* ∈ *V* if *c* is found to be significantly associated with the expression of *v* in an expression quantitative trait loci (eQTL) screen. We refer to these as *eQTL edges*.
- *E* contains an edge between two genes *u* and *v* if *u* and *v* code for interacting proteins. We refer to these as *PPI edges*.

Note that Azencott *et al.* [30] also propose the idea of integrating multiple types of networks to drive the search for phenotype-associated genomic loci. However, the heterogenous network model proposed here encapsulates more biological information in a sparser network by allowing nodes and edges to represent different types of biological entities and interactions/associations. Moreover, the incorporation of eQTL links in the network makes this method particularly powerful since these links capture functional associations also for loci that are outside coding regions or RoIs of genes.

**Fig 3:**
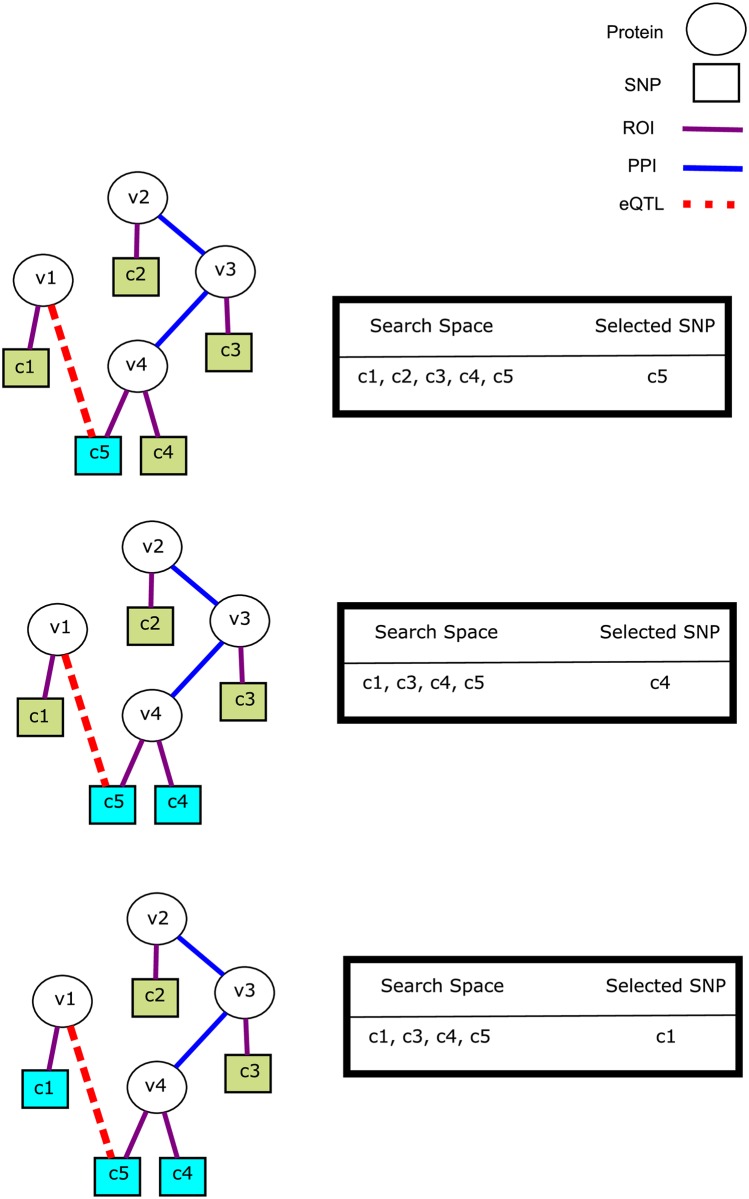
Identification of NetPocos. Each *v*_*i*_ represents a protein (*V*) and each *c*_*j*_ represents a genomic locus (*U*). Blue edges represent the interactions between proteins (*E*), purple edges indicate that the respective locus is in the RoI of the coding gene for the respective protein and red edges represent the eQTL links. Initially, *P* is empty and all loci are considered and the locus (*c*_5_) that maximizes *δ*(.) is added to *P*. After this point, the search space is restricted to loci that are at most three hops away from *c*_5_. We continue this procedure until the set of selected loci cover a sufficient fraction of the case samples. Cyan nodes and gold nodes show the selected loci and proteins respectively.

The algorithm for identifying NetPocos is illustrated in Fig 3. This algorithm proceeds similarly to the algorithm for identifying network-free PoCos. However, while growing PoCos, the set of loci that can be added to a growing PoCo *P* is constrained by the network. Namely, at any step of the algorithm, only loci that are at most 3 hops away from at least one locus in *P* are considered as candidates for addition into *P*. This ensures that the loci in a NetPoco are functionally related to each other. In other words, reachability within three hops captures all functional association patterns between a pair of loci in this heterogeneous network:

- ROI-ROI association: Two loci that are in the RoI of the same gene are within 2 hops of each other.
- ROI-eQTL association: A locus that is in the RoI of a gene *u* is 2 hops away from loci that are associated with *u*’s expression.
- ROI-PPI-ROI association: Two loci that are in the RoI of the genes coding for two interacting proteins are within 3 hops of each other.
- ROI-PPI-eQTL association: A locus that is in the RoI of a gene *u* is 3 hops away from a locus that is associated with the expression of gene *v* such that the products of *u* and *v* interact with each other.

When the algorithm terminates, it returns the set *Π* of all discovered PoCos. As we discuss in the next section, each identified PoCo contains multiple loci and most of the loci in the dataset are not assigned to any of the PoCos in practice. For this reason, we usually have |*Π*| < < |*C*|.

### Model Development for Risk Assessment

One potential utility of the PoCos is risk assessment. By construction, PoCos (NetPocos) contain (functionally associated) loci that exhibit improved power in distinguishing cases from control. Consequently, as compared to individual variants, they may provide more robust and reproducible features to be used in predictive models. To investigate the utility of these multi-locus features in risk assessment, we use PoCos to build a model for risk assessment using L1 regularized logistic regression classifier.

#### Representative genotypes of PoCos

To facilitate the use of PoCos for risk assessment, we compute a representative genotype for each PoCo. For this purpose, we use the fraction of the loci in the PoCo that harbor a genotype of interest in the respective sample. To be more precise, for each PoCo *P* ∈ *Π*, we compute the profile of *P* as

$$h\left(P,s\right)=\frac{\sum_{c\in P}M(c,s)}{\left|P\right|}$$ (12)

for all *s* ∈ *S*. The set of features utilized by the classifier is comprised of *h*(*P*,.) for all *P* ∈ Π. Next, we perform feature selection to identify a parsimonious set of PoCos to be used in risk assessment.

#### Feature selection and model building

High dimensionality is always an important problem in GWAS (a.k.a. “large p small n”). The large number of features makes feature selection quite challenging. In particular, the models can be easily over-fit if too many features are entered into the model. For this reason, many researchers suggest filtering algorithms for dimension reduction and feature selection [40–42]. Furthermore, building the L1-regularized logistic regression model is computationally expensive, and reduction in the number of features can greatly reduce runtime. Motivated by these considerations, to find the optimal set of PoCos to be used for risk assessment, we use a two-step feature selection method. The first step implements filtering-based feature selection, and the second step incorporates feature selection into model building by using a L1-regularized logistic regression classifier that enforces sparsity. Note that feature selection is applied within a cross-validation framework, so that test samples are not used in the identification and selection of the PoCos that are used in the model.

For filtering-based feature selection, we compute a *p*-value representing the significance of the association of each PoCo with the disease. For this purpose, we use two different methods:

- **Logistic regression:** We compute a logistic regression model by including all identified PoCos in the model. The *p*-value of the coefficient of each PoCo in this model represents the significance of the PoCo in predicting phenotype at the presence of all other PoCos.
- **KS-statistic:** We assess the significance of the Kolmogorov-Smirnov (KS) statistic comparing the distribution of *h*(*P*, *s*) in case samples against that in control samples. The *p*-value of the KS-statistic quantifies the significance of the difference between the two sample classes in terms of the distribution of the values of the feature representing that PoCo.

We then apply a threshold on these *p*-values to reduce the number of PoCos that are used in model building. Namely, for a given threshold *α*, we filter out all PoCos with *p*-value greater than *α* and retain all other PoCos to be entered into model building. This is done separately for each of the filtering methods.

Let *H* be the matrix in which rows represent samples and columns represent PoCos that pass the filtering stage, such that *H*(*s*, *p*) = *h*(*p*, *s*). As before, *f* denotes the vector composed of the phenotypes of samples. Then the L1-regularized logistic regression classifier computes a vector *β* to solve the following optimization problem:

$$\min_{\beta \in R^{q}}\left\{-log\;p\left(f|H;\beta \right)+{\lambda ∥\beta ∥}_{1}\right\}$$ (13)

Here, *q* denotes number of PoCos that are entered into the model and *λ* is a non-negative regularization parameter. The second term in the objective function is a penalty function that enforces sparsity of the model and the parameter *λ* controls the number of PoCos selected in the model (i.e., the number of non-zero entries in *β*). For larger *λ*, the model is expected to be more sparse.

#### Performance evaluation for risk assessment

To evaluate the performance of PoCos in risk assessment, we use nested *K*-fold cross validation. Namely, we divide the set of samples into *K* subsets {*T*_1_, …, *T*_*K*_}, while keeping the proportion of case and control samples fixed across all subsets. For the *k*th subset of samples, we reserve the samples in this subset as test samples. We divide the training group further into *K* groups and use this partitioning to perform genotype identification, PoCo identification, feature selection, filtering-based feature selection and model building using the L1-regularized logistic regression classifier. Once the model is optimized in the inner fold, we use the resulting model to predict the class of each sample in the *k*th subset, and evaluate prediction performance on this outer fold. This process is iterated for *k* = 1, 2, …, *K* and the performance of classification is evaluated based on the predictions across all samples. The typical choices of *K* are 5 or 10 and here we use 5-fold cross validation in our experiments. We also repeat the randomization of folds five times and report the averages of performance figures across these randomizations.

Risk assessment models produce quantitative predictions of susceptibility to the disease of interest. To evaluate the predictive ability of these risk assessment models, we apply different thresholds on the predicted risk to obtain a binary prediction for each test sample. Using these binary predictions, we obtain the counts of true positives (predicted to be in risk, has the disease), false positives (predicted to be in risk, does not have the disease), and false negatives (predicted not to be in risk, has the disease), and compute the precision (fraction of true positives among all predicted to have risk) and recall (fraction of true positives among all who have the disease) figures based on these counts. We assess the performance of each risk assessment model based on the area under the ROC curve (AUC), which characterizes the ability of the model in trading off precision and recall for varying thresholds on the quantitative prediction.

#### Polygenic score

We compare the performance of PoCo-based risk assessment models against models based on individual loci, as well as Polygenic score. Polygenic score is a commonly used method for risk assessment in GWAS. It is based on the assumption that the joint effect of multiple loci on the phenotype is additive [20]. Based on this assumption, the polygenic score for an individual is defined as the summation of the effect sizes of multiple loci, weighted by effect sizes of individual loci. To estimate effect sizes, the *p*-value of the association of each loci with the phenotype is calculated. For a given parameter *α*, *L* is defined as the set of loci with *p*-value less than *α*. Subsequently, the polygenic score for a sample *s* is the defined as follows:

$$PS_{\alpha }\left(s\right)=\sum_{c\in L}\gamma \left(c\right)*g\left(c,s\right)$$ (14)

Here, *γ*(*c*) denotes the effect size of locus *c* which can be estimated using an appropriate regression model(i.e. logistic for a binary phenotype or linear for a continuous phenotype).

## Results

To assess the ability of PoCos in producing informative multi-locus features, we evaluate their utility in the context of risk assessment. For this purpose, we use GWAS data from the Welcome Trust Case-Control Consortium (WTCCC), which includes data from studies for seven complex diseases, namely type 1 diabetes (T1D), type 2 diabetes (T2D), psoriasis (PS), bipolar disorder (BD), coronary artery disease (CAD), hypertension (HT), and multiple sclerosis (MS). On each dataset, we first identify PoCos, select features to build a model for risk assessment, and then evaluate the performance of the resulting model. To control for overfitting and to ensure that the performance figures are not biased, we use cross validation.

We first compare the risk assessment performance of the multi-locus features against the standard approach of using individual significant loci. To facilitate fair comparisons, we use the classification and feature selection methods described in the “performance evaluation for risk assessment” section identically for all types of multi-locus and individual-locus based features. We also compare the performance of NetPocos against *Polygenic Score*, which is a commonly used method for risk assessment. Subsequently, to gain insights into the information provided by network data and specifically eQTL-based regulatory interactions, we also compare the performance of NetPocos, network-free PoCos, and eQTL-free PoCos. Moreover, we investigate the effect of *λ* in the L1 regularized logistic regression classifier, i.e. the parameter that controls the parsimony of the model. We also assess the biological relevance of some of the selected PoCos using enrichment analysis and a literature-driven list of genes and processes that have been reported to be associated with diseases. Finally, we compare the most frequently recruited genes in PoCos in different diseases to gain insights into shared genetic bases of different diseases. This analysis also suggests novel potential susceptibility genes for these diseases.

### Experimental Setup

#### GWAS datasets

We use genome wide association data for all seven diseases obtained from the Wellcome Trust Case-Control Consortium (WTCCC) [43–45]. For each dataset, we use the genotypes generated by Chiamo algorithm. We filter out the loci with minor allele frequency (MAF) ≤ 5%. While identifying the PoCos, in order to avoid marginal effect of individual loci and reduce the risk of artifacts, we filter the loci with nominal p-value of individual association less than ≤ 10^−7^ (this corresponds to a corrected p-value threshold of 0.05). Since we utilize the PPI networks and eQTL data to identify NetPocos, we include in our analyses the SNPs that are either within 50*kb* upstream and downstream of coding regions or are identified by eQTL to be associated with the expression of a gene. The number of loci and the the number of samples for each dataset are shown in Table 1.

**Table 1:** Genome-Wide Association data used in the computational experiments.

	T1D	BD	HT	T2D	PS	CAD	MS
Number of genotyped loci	385134	330651	372461	409022	531592	322148	9469
Number of loci in network	248669	214464	248868	258217	255494	239763	8267
Number of control samples	2997	2997	2997	1504	5175	2997	2930
Number of case samples	2000	1998	2001	1999	2178	1988	975

#### Protein-protein interaction (PPI) dataset

We use a human PPI network downloaded from BioGRID (The Biological General Repository for Interaction Datasets) database. The BioGRID PPI network contains 194639 interactions among 18719 proteins.

#### Expression quantitative trait loci (eQTLs) datasets

We use an eQTL dataset obtained from RegulomeDB which aims to annotate noncoding common variants from association studies [46]. This database contains high throughput datasets from The Encyclopedia of DNA Elements (ENCODE) [47] and other resources, as well as computational prediction and manual annotation. We extract all the variants that are identified to have direct effect on gene expression and also have been shown to be on transcription binding sites through ChIP-seq and DNase with either a matched PWM to the ChIP-seq factor or a DNase footprint.

#### SNP-gene mapping

To identify network-free PoCos, we do not use gene information. To facilitate the identification of NetPocos, we map SNPs to genes by defining the region of interest (RoI) for a gene as the genomic region that extends from 50kb upstream to 50kb downstream of the coding region for that gene.

#### Association analysis for individual loci

We identify individually significant loci using PLINK [48], a well-established toolkit for GWAS analysis. We assess the disease association of all loci in each dataset based on minor allele frequency, obtaining a p-value for the association of each locus with the disease. We adjust the p-values for multiple hypothesis testing using Bonferroni correction.

### Performance of PoCos in Risk Assessment

For each dataset, we divide the population into 5 groups while preserving the proportion of case and control samples in each group. We reserve one group for testing and we identify NetPocos on the remaining four groups. Then, we use these four groups for feature selection and model building. Finally, we test the performance on the group reserved for testing. All of the reported performance figures are averages across five different cross-validation runs. The number of PoCos identified on each dataset and the size of these PoCos are presented in Table 2. Please note that the variance in number of PoCos does not have a significant effect on the performance (S1 Fig).

**Table 2:** The number of PoCos identified on each dataset, and the distribution of the genomic loci in each individual PoCo.

	T1D	BD	HT	T2D	PS	CAD	MS
Number of PoCos	19867±14268	16542±1074	5300±7865	8147±5791	23959±9424	8474±3937	243±111
Number of SNPs per PoCos	2.99±0.99	3.42±0.85	3.34±0.88	3.72±0.67	3.5±0.72	3.48±0.76	3.05±0.6

The average and standard deviation is reported across different folds.

#### Comparison of NetPocos against individual loci and polygenic score

To investigate the benefits of using NetPocosin risk assessment, we first compare the performance of NetPoco-based risk assessment models against that of individual-locus based models and the well-established Polygenic Score. As described in the Methods section, we select NetPocos to be used in model building using a filtering based feature selection method, which uses *p*-values (of either the coefficient in logistic regression model or the KS-statistic for difference in the distribution between case and control samples) as the filtering criterion. Similarly, we filter individual loci based on the statistical significance of their association with the disease (after correction for multiple hypothesis testing). Polygenic risk score, which is commonly used in risk assessment, is a sum of the scores of associated loci, weighted by effect sizes, which are estimated using the training set. For polygenic score, the features are also selected using the *p*-value threshold in training samples and they are used to score the individuals in test samples.

To comprehensively understand the effect of filtering, we test all methods using different thresholds on p-value for filtering (*α*). Namely, for each *α* ∈ {5*E* − 8, 0.05, 0.1, 0.15, 0.2, 0.25, 0.3}, we build the risk assessment model using the NetPocos or loci with *p*-value less than *α*. Note that we use p-values to rank and select individual loci or PoCos to be entered into the model as features. As discussed in Methods, the *p*-values for PoCos reflect the significance of logistic regression coefficients or KS-test, whereas for individual loci, the *p*-value reflect the significance of case/control association analysis as computed by PLINK. Since *p*-value are used for ranking, correction for multiple hypotheses does not influence the behavior of the methods. Nevertheless, the *p*-value thresholds shown in the figure are based on Bonferroni-corrected *p*-value. For model building, we use the L1 regularized logistic regression classifier described in the Methods section, for both NetPocos and individual locus based features. L1 regularized logistic regression provides a second layer of feature selection through the regularization term in the associated objective function. Polygenic score has its own classification algorithm by definition.

**Fig 4:**
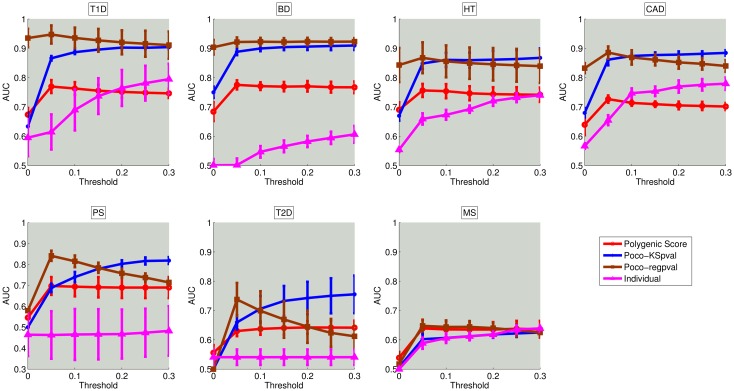
Comparison of the risk assessment performance of NetPocos, individual locus based features, and polygenic score on seven different diseases. The x-axis shows the *p*-value threshold (*α*) used in filtering based feature selection and the y-axis shows the area under the ROC curve (AUC) for performance in risk assessment. The curve shows the average AUC score and error bars show the standard deviation of AUC score across 5 folds in 5 different runs.

The results of cross-validation for using individual loci (using L1 regularized logistic regression), polygenic score, and NetPocos with two different filtering criteria (logistic regression p-values vs. KS-statistic) are shown in Fig 4.

The results shown in Fig 4 suggest that filtering of NetPocos based on regression *p*-value provides favorable prediction performance when a strict threshold is used for statistical significance (i.e., for smaller *α*). However, as the threshold increases (i.e., more NetPocos are entered into model building), the performance of regression based filtering declines. On the other hand, the prediction performance of NetPocos filtered based on KS *p*-value is improved with increasing threshold on significance. This observation suggests that, while regression *p*-value tends to rank the most informative NetPocos at the top, KS-statistic based ranking provides a more reliable set of NetPocos for L1 regularized logistic regression to choose from when more NetPocos are entered into the model (S2 Fig).

Comparison of Polygenic Score and NetPoco-based risk assessment in Fig 4 shows that NetPoco-based models consistently outperform Polygenic Score for all diseases, perhaps with the exclusion of multiple sclerosis. Overall, Polygenic Score has a peak performance at relatively stricter thresholds on the significance of individual loci included in the model, but this figure remains under the peak performance of NetPoco-based models. Individual locus based classifier performs more favorably when more loci are entered into the model (which is expected since L1 regularized logistic regression effectively performs feature selection), but the performance of the classifier that uses individual locus based features remains below the performance of the classifier that uses NetPoco-based features. These results suggest that NetPocos are useful in “feature construction” for risk assessment, i.e., they bring together robust sets of loci to be used together in risk prediction (S3 Fig). It is also possible that, as compared to using standard genotype coding for individual loci, our method for computing representative genotypes for PoCos improves prediction performance, since it potentially captures non-linear relationships among PoCos as well.

**Fig 5:**
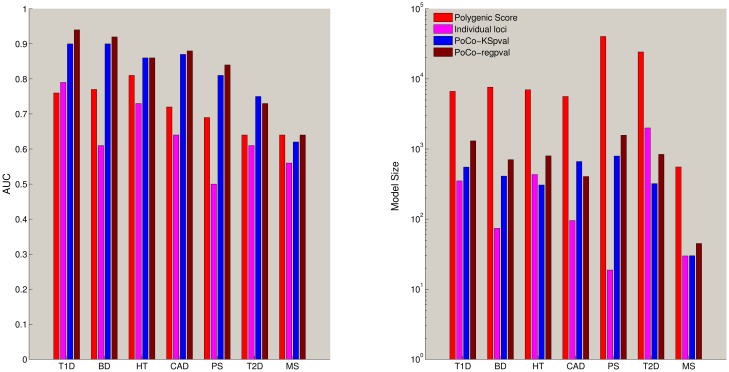
The best risk prediction performance achieved by each method and the size of the resulting model for all seven diseases.

To facilitate thorough comparison of NetPocos, individual locus based features, and Polygenic Score, we also report the best average AUC and the number of features in the final model across all p-value thresholds used for filtering. These results are shown in Fig 5. As seen in the figure, models that use NetPoco-based features consistently outperform individual locus based features and Polygenic Score in risk assessment for all diseases, and they provide more parsimonious models as compared to Polygenic Score. However, it is interesting to note that PoCoS do not provide significant improvement in risk assessment for MS. This is the dataset that has the smallest number of loci. To this end, this behavior may be indicative of the need for higher coverage to be able to identify more informative PoCos.

#### NetPocos vs. network-free PoCos

**Fig 6:**
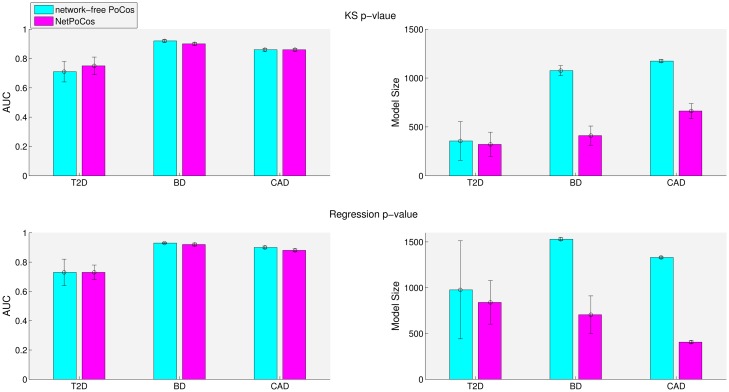
Comparison of the risk assessment performance of NetPocos and network-free PoCos on T2D, BD and CAD using KS *p*-value (first row) and regression *p*-value (second row). The colored bars show the average AUC score and the error bars shows the standard deviation of AUC score across the folds.

Many computational methods are developed to integrate the GWAS data with other biological datasets that provide information on the functional relationships between individual biological entities (here, genomic loci). In this study, we integrate PPI data and eQTL data in the identification of NetPocos. Since the identified NetPocos are guided by the PPI network and eQTL data, we expect that NetPocos would be more informative and robust as compared to network-free PoCos, since they are composed of functionally related loci. To investigate whether this hypothesis is supported empirically, we compare the performance of NetPocos in risk assessment to that of network-free PoCos. For this purpose, since the computation of network-free PoCos is computationally expensive, we limit our analyses to three diseases: bipolar disorder (BD), type II diabetes (T2D), and coronary-artery disease (CAD). The results of these analyses are shown in Fig 6. Note that, in these analyses, network-free PoCos have been identified using all genotyped loci and the search space is not limited to the loci that can be mapped to gene regions. Therefore, network-free PoCos can include some loci that are out of gene regions as well, providing them with an advantage over NetPocos. However, as seen in the figure, NetPocos outperform the network-free PoCos for T2D. In contrast, the results for BD and CAD suggest that constraining the search space by functional interactions based on PPIs and eQTL may slightly reduce the predictive power of PoCos. However, importantly, when we consider model size, we observe that NetPocos provide more parsimonious final models for all three diseases.

**Fig 7:**
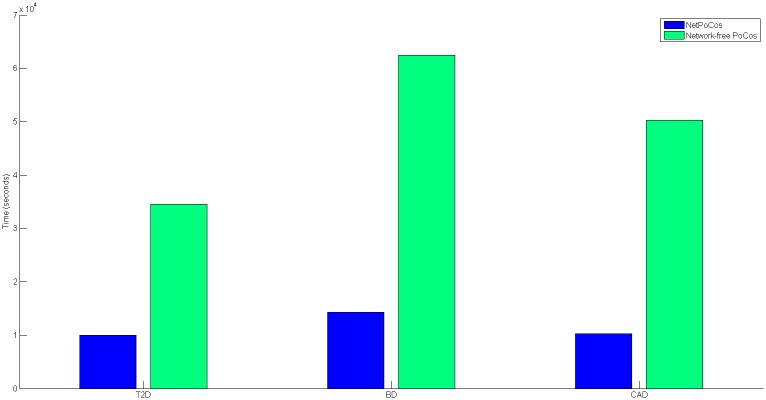
Comparison of the running time for identification of NetPocos and network-free PoCos on T2D, BD and CAD.

We implement the procedure for the identification of PoCos in MATLAB. We assess the runtime of this procedure using Intel(R) Xeon(R) CPU E5-4620 with a 2.2 GHz processor with 50 GB RAM. The results of this analysis are shown in Fig 7. These results suggest that incorporating interactions among proteins and eQTL data can effectively improve the quality of PoCos by providing more parsimonious models. Furthermore, using prior knowledge makes the problem computationally feasible since it drastically reduces the running time.

#### Information added by eQTL data

An important limitation of network-based analyses of GWAS data stems from the constraints posed by the lack of regulatory interactions in network models. If the functional relationships that are used to drive the search are limited to protein-protein interactions (PPIs), the search is limited to loci that are in close proximity to coding regions and regulatory interactions that involve non-coding loci are not considered [31]. One important contribution of this study is the incorporation of eQTL-based interactions along with PPIs to drive the search for NetPocos. To assess the benefits of including eQTL-based interactions, we also identify PPI-based PoCos using a network that does not contain eQTL edges, and compare the risk assessment performance of these PoCos against that of NetPocos (which are identified using PPI and eQTL data). Note that removal of eQTL edges causes the removal of loci that are connected to the network just by eQTL edges. Such loci are usually those that are not in close proximity of coding regions.

**Fig 8:**
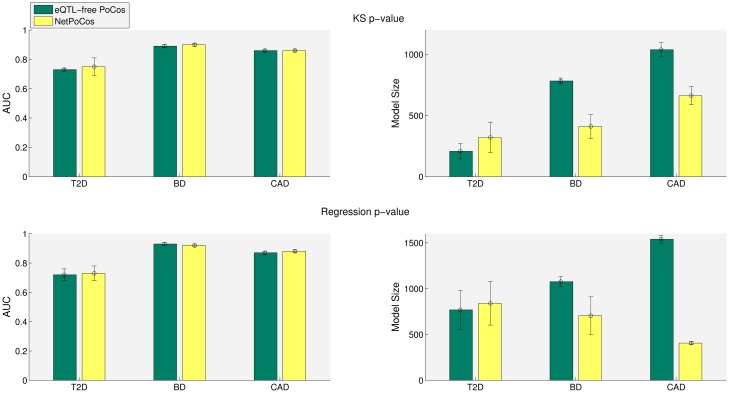
Comparison of the risk assessment performance of NetPocos (which include eQTL-based regulatory interactions and PPIs) and eQTL-free PoCos (which contain PPIs only) on T2D, BD and CAD. PoCos are filtered based on KS *p*-value (first row) and regression *p*-value (second row). The colored bars show the average model size and AUC score and the error bars show the standard deviation of these figures across 5 runs.

The results of this analysis are presented in Fig 8. As see in the figure, the performance of PPI-only PoCos and eQTL+PPI-based NetPocos is similar for all three diseases. However, for BD and CAD, the predictive models provided by the incorporation of eQTL data are significantly more parsimonious than the models provided by PPI-only NetPocos. For T2D, the incorporation of eQTL edges leads to more complex models, but the prediction performance is enhanced with the inclusion of eQTL edges. These observations suggest that incorporation of eQTL data indeed provides biologically relevant information in the discovery of NetPocos.

#### Effect of model complexity

**Fig 9:**
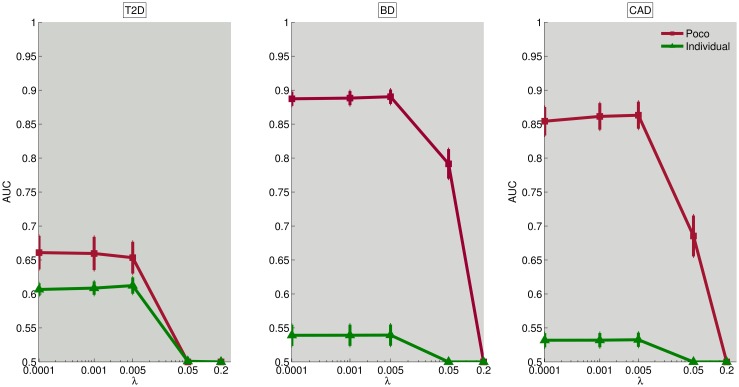
Effect of model sparsity on the prediction performance of L1 regularized logistic regression classifier using individual SNPs vs. NetPocos. The x axis shows *λ*, which is the coefficient of penalty function in L1 regularized logistic regression, and y axis shows the cross-validation performance of the model (AUC) across 5 runs.

In L1 regularized logistic regression, the parameter *λ* in Eq 13 is used to tune the trade-off between model fit and model complexity (number of features included in the model). Larger *λ* forces the model to be more parsimonious. Therefore, as *λ* grows, the learning task becomes more difficult, in that L1 regularized logistic regression tries to simplify the model by compromising model fit. For this reason, if the features that are input into the classifier are “high-quality” features, the classifier can be expected to be more robust to this parameter. Based on this premise, we assess the “quality” of the features constructed from NetPocos by comparing the models based on NetPocos and individual loci in terms of their performance as a function of *λ*. For this purpose, we fix the *p*-value threshold (0.05) for both NetPocos and individual SNPs and compute the AUC in cross-validation for a range of different values of *λ*. The results of this analysis are shown in Fig 9. As seen in the figure, as lambda gets larger, the risk assessment performance of individual loci quickly becomes equivalent to that of a coin toss. This observation suggests that the classifier needs to incorporate a large number of features to maintain model fit, which may make the classifier vulnerable to overfitting. This is also true for NetPocos, but NetPocos can tolerate larger lambdas.

In all other results reported in this section, we use *λ* = 0.001 which provides a reasonable balance between the complexity and predictive performance of the model.

#### Biological interpretation of NetPocos

We assess the biological relevance of the predictive NetPocos using pathway analysis, Gene Ontology enrichment analysis, and literature-driven list of genes and processes that are reported to be associated with disease. For this analysis, we focus on three diseases (T2D, CAD and BD) which are shown to have similar molecular mechanisms [49, 50] and share common risk pathways [51].

**Fig 10:**
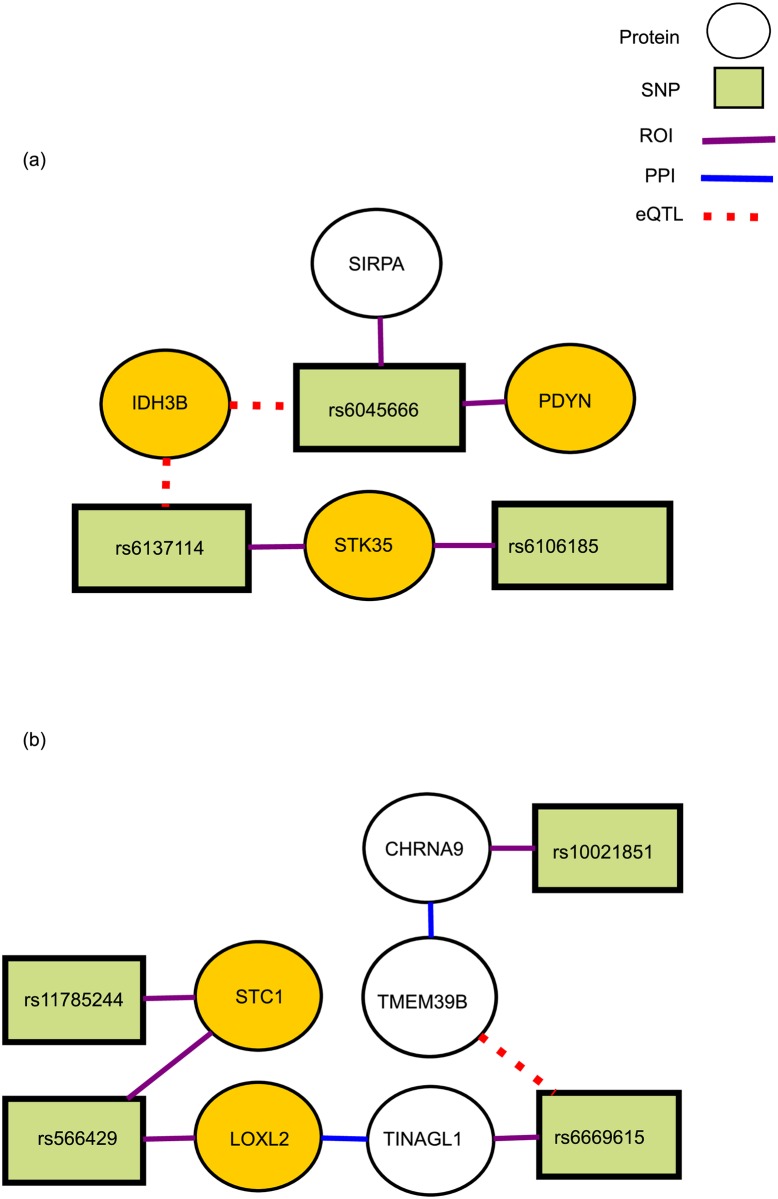
Two NetPocos associated with T2D. Two NetPocos associated with Type II Diabetes (T2D). These NetPocos are consistently selected by L1 regularized logistic regression in the final model for risk prediction. The circle nodes represent proteins and rectangular nodes represent SNPs. Red dashed lines represent the eQTL association between a SNP and a gene, purple lines indicate that a SNP is in the ROI of the respective gene, and the blue edges represent a protein-protein interaction (PPI) between the products of respective genes. The genes that are previously reported to be associated with T2D are highlighted in gold. (a) A NetPoco enriched in isocitrate metabolic process and *NADH* metabolic process(*p*-value = 0.004), which both contribute to the amplification of insulin, (b) a NetPoco enriched in calcium ion homeostasis.

*Type II Diabetes (T2D).* We focus on NetPocos that have highest coefficient in the model constructed by L1-regularized logistic regression classifier. Top two NetPocos are shown in Fig 10. The NetPoco shown in Fig 10(a) induces a subgraph that does not contain any PPI edges. However, eQTL edges are able to capture the functional relationship between the SNPs and the genes in this NetPoco. Interestingly, some of the genes in this NetPoco are previously reported to be associated with T2D [52], while some may have links to T2D although no direct associations are previously reported. More precisely, Wong *et al.* [53] show that SIRPA is a T1D risk gene in the non-obese diabetic mouse. The inclusion of this gene in a NetPoco that is used in risk assessment for T2D suggests that this gene can be a potential novel candidate for association with T2D as well. We also use ontologizer for Gene Ontology enrichment analysis [54]. The Gene Ontology enrichment analysis shows that this PoCo is enriched in isocitrate metabolic process (*p*-value = 0.001) and also *NADH* metabolic process(*p*-value = 0.004), which both contribute to the amplification of insulin secretion [55].

The PoCo shown in Fig 10 contains both PPI and eQTL-based edges. *STC1* and *LOXL2* are genes that are previously reported to be associated with T2D [52]. It is notable that *TINAGL1* is involved in Glucose/Energy metabolism pathway and *CHRNA9* is involved in Postsynaptic nicotinic acetylcholine receptors pathway with other genes such as *CHRNA2*, *CHRNA4* and *CHRNA6* that are previously reported to be associated with T2D [52]. This observation suggests that *TINAGL1* and *CHRNA9* can be potential candidate genes for T2D. Additionally, it is known that acetylcholine can enhance glucose-stimulated insulin secretion from pancreatic beta-cells [56]. This PoCo is also enriched in calcium ion homeostasis (*p*-value = 0.001) which is one of the T2D associated pathways.

Note that, for T2D, non-genetic risk factors including age, sex, and body-mass index (BMI) play an important role in risk. These factors can be also combined with genetic factors to obtain better performance in risk assessment [57]. Janipalli et al. [58] combine 32 genomic loci with other conventional risk factors to obtain an AUC of 0.63 in an Indian population. Therefore the performance improvement provided by the multi-locus features as compared to the individual locus based features in a genetic factor only setting suggests that combination of multi-locus genomic features with other factors may lead to an even greater predictive performance in risk assessment.

**Fig 11:**
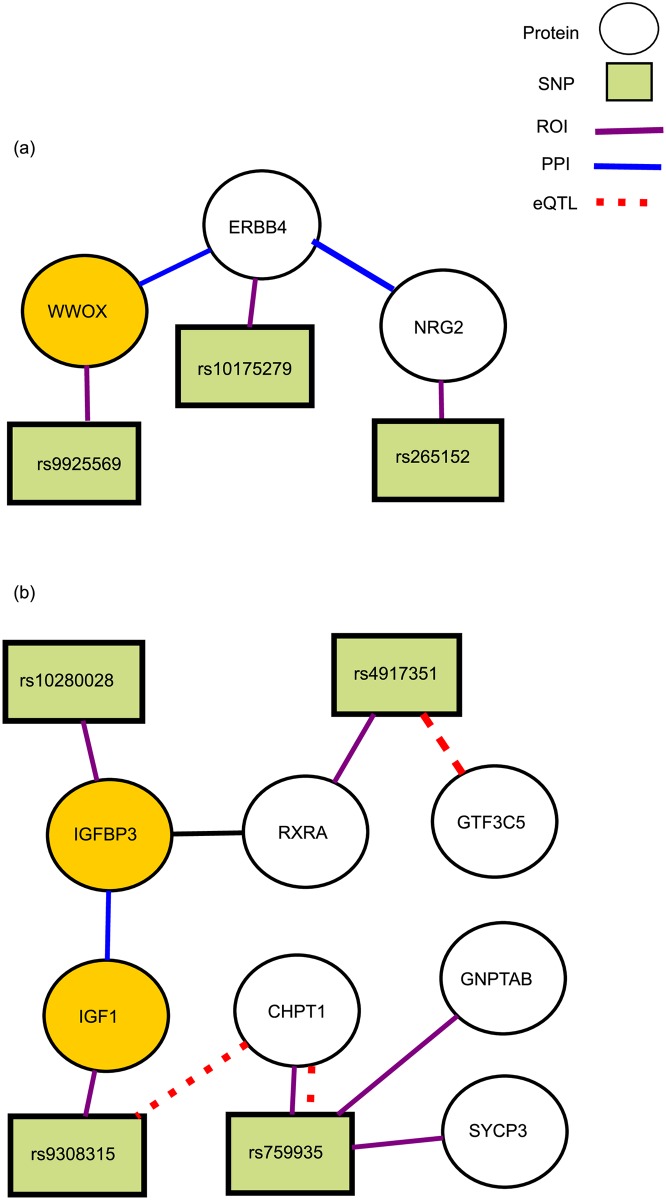
Two NetPocos associated with CAD. Two NetPocos associated with Coronary Artery Disease (CAD). These NetPocos are consistently selected by L1 regularized logistic regression in the final model for risk prediction. The circle nodes represent proteins and rectangular nodes represent SNPs. Red dashed lines represent the eQTL association between a SNP and a gene, purple lines indicate that a SNP is in the ROI of the respective gene, and the blue edges represent a protein-protein interaction (PPI) between the products of respective genes. The genes that are previously reported to be associated with CAD are highlighted in gold. (a) A NetPoco enriched in cardia muscle tissue regeneration (*p*-value = 0.0003) and activation of MAPKK activity (*p*-value = 0.02), (b) a NetPoco enriched in positive regulation of insulin-like growth factor receptor signaling pathway (*p*-value = 0.0006).

*Coronery-Artery Disease (CAD).* Two NetPocos that have highest coefficient in L1 regularized logistic regression for CAD are shown in Fig 11. The genes that are highlighted in gold code for proteins that are previously reported to be associated with CAD [59]. The NetPoco in Fig 11(a) is enriched in positive regulation of STAT protein (*p*-value = 0.0003), positive regulation of cardiac muscle cell proliferation (*p*-value = 0.002), cardiac muscle tissue regeneration (*p*-value = 0.0003), and activation of *MAPKK* activity (*p*-value = 0.02). These pathways are previously reported to be associated with susceptibility to CAD [59]. Although *ERBB4* is not previously reported to be associated with CAD, it plays a role in *MAPK* pathway, which is one of the top pathways for CAD [59]. Therefore, *ERBB4* can be a potential candidate gene for CAD as well. The NetPoco in Fig 11(b) is also enriched in muscle cell proliferation (*p*-value = 3.3E-6), prostate glanduar acinus development (*p*-value = 5.92E-6), and muscle cell differentiation(*p*-value = 5.72E-5). This NetPoco is also enriched in positive regulation of calcieneurin-NFAT signaling pathway (*p*-value = 0.0006) and positive regulation of insulin-like growth factor receptor signaling pathway. *IGF1* and *RXRA* are both involved in a pathway named “Pathways in cancer” which is known to be related to CAD. More than 20 genes in this pathway are known to be associated with CAD [59]. This observation suggests that *RXRA* may be a novel CAD risk factor.

**Fig 12:**
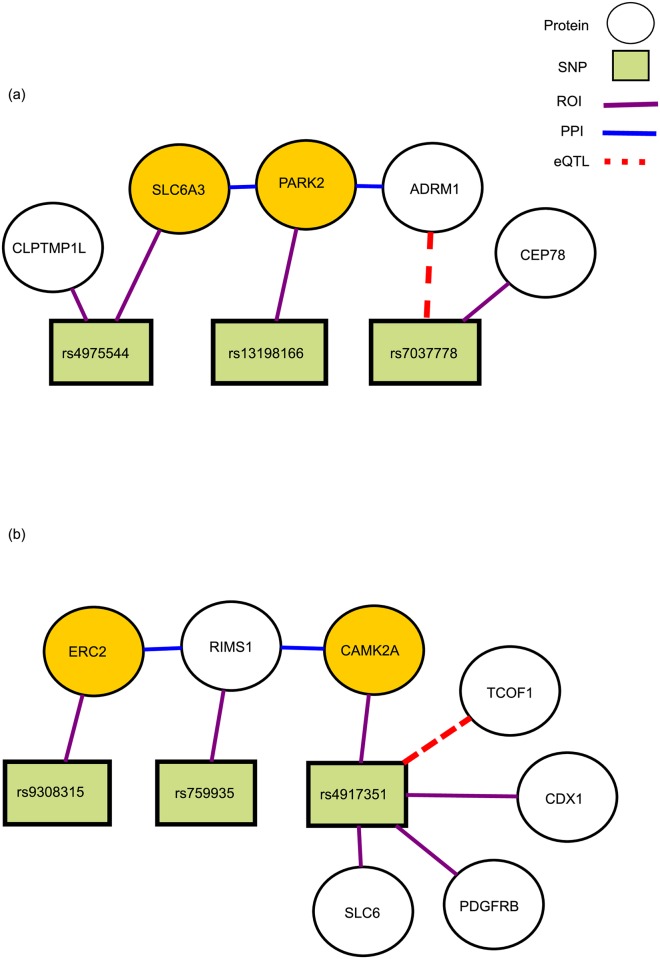
Two NetPocos associated with BD. Two NetPocos associated with Bipolar Disorder (BD). These NetPocos are consistently selected by L1 regularized logistic regression in the final model for risk prediction. The circle nodes represent proteins and rectangular nodes represent SNPs. Red dashed lines represent the eQTL association between a SNP and a gene, purple lines indicate that a SNP is in the ROI of the respective gene, and the blue edges represent a protein-protein interaction (PPI) between the products of respective genes. The genes that are previously reported to be associated with CAD are highlighted in gold. (a) A NetPoco enriched in regulation of dopamin metabolic process which plays a central role in bipolar disorder (*p*-value = 8.67e-6), (b) a NetPoco enriched in regulation of neurotrasmitter secretion and insulin receptor signaling pathway (*p*-value = 0.0007).

*Bipolar Disorder (BD).* Two NetPocos with highest coefficient in L1 regularized logistic regression in the risk prediction model for bipolar disorder are shown in Fig 12. TheNetPoco in Fig 12(a) is enriched in regulation of dopamin metabolic process (*p*-value = 8.67e-6), which plays a central role in bipolar disorder [60]. The NetPoco in Fig 12(b) is enriched in regulation of neurotrasmitter secretion (*p*-value = 0.0007), cell migration involved in coronary angiogenesis (*p*-value = 0.0008), and insulin receptor signaling pathway(*p*-value = 0.003).

*Shared molecular bases among diseases.* Identifying the links between the molecular etiologies of different diseases can provide an insights on the underlying mechanisms of these diseases. Elucidation of such relationships can also help to detect the novel candidate genes for diseases. For example, patients with bipolar disease frequently have coexisting medical conditions such as obesity, cardiovascular disease, and diabetes mellitus [49]. Torkamani *et al.* [50] also show a strong genetic correlation between BD and metabolic disorders CAD and T2D. Note that the results of Gene Ontology enrichment analysis reported above also suggest that NetPocos can capture the relationship between diseases. For example, the NetPoco in Fig 11(b), which is associated with CAD, is enriched in regulation of insulin-like growth factor receptor signaling pathway, which is also associated with T2D.

To gain further insights into the shared molecular bases of T2D, CAD, and BD, we examine the genes that appear most frequently in the NetPocos selected by L1 regularized logistic regression in the risk assessment models for these diseases. For each disease, we identify the top 10 most frequent genes. We then assess whether they are previously reported to be associated with T2D [52], CAD [59] and BD [61, 62] as well. The results of this analysis are shown in Table 3. The first ten rows show the most frequent genes in NetPocos identified in CAD dataset. Among these genes, *WWOX* and *CD36* are previously reported candidates for CAD. They are also known to be associated with BD. This result suggests that, for example, *GRID1* can also be a potential susceptibility gene for CAD. This hypothesis is also supported by the observation that *GRID1* plays a role with 14 other known CAD genes in neuroactive legand-receptor interaction. *WWOX* also can be a good candidate for T2D, considering that it plays a role in apoptosis and autophagy pathway, which is the main form of beta-cell death in T2D [63].

**Table 3:** Shared molecular bases of T2D, BD, and CAD as revealed by NetPocos.

Gene Name	Frequency	T2D	BD	CAD
CAD				
*WWOX*	102	NO	YES	YES
*CSMD1*	80	NO	YES	NO
*APP*	77	NO	NO	NO
*PARK2*	74	YES	YES	NO
*GRID1*	64	YES	YES	NO
*DOCK10*	61	YES	NO	NO
*CUL3*	56	NO	NO	NO
*DENND1A*	56	NO	NO	NO
*CD36*	52	NO	YES	YES
*CNTNAP2*	49	NO	YES	NO
T2D				
*CSMD1*	38	NO	YES	NO
*A2BP1*	35	NO	YES	NO
*FHIT*	35	NO	NO	NO
*CNTNAP2*	34	NO	YES	NO
*PTPRD*	32	YES	YES	NO
*CACNA2D3*	31	NO	NO	NO
*WWOX*	29	NO	YES	YES
*NRG1*	28	YES	YES	YES
*SUPT3H*	28	YES	NO	NO
*CDH13*	26	NO	YES	YES
BD				
*PARK2*	54	YES	YES	NO
*NRG1*	48	YES	YES	YES
*ADRB2*	47	YES	YES	YES
*WWOX*	43	NO	YES	YES
*APP*	41	NO	NO	NO
*CACNA2D3*	40	NO	NO	NO
*CUL3*	40	NO	NO	NO
*KIF16B*	40	NO	NO	NO
*SNX29*	35	NO	NO	NO
*DAPK1*	33	NO	NO	NO

For each disease, ten most frequent genes that are involved in NetPocos selected by L1 regularized logistic regression in risk prediction are listed. Previously reported association of these genes with the three diseases are indicated with a “Yes” or “No” in the respective column of each row.

Note that NetPocos do not overlap at the SNP-level, however, they may overlap at the gene-level since multiple SNPs can be mapped to the same gene. This shows the power of NetPocos in identifying molecular bases of diseases, since multiple NetPocos can arise from similar functional contexts, providing stronger statistical evidence for the involvement of genes that are associated with these NetPocos.

## Discussion

In this paper, we propose a novel criterion to assess the collective disease-association of multiple genomic loci (PoCos) and investigate the utility of these multiple-loci features in risk assessment. We also perform extensive experiments to evaluate the effect of using network information to drive the search for multi-locus features on risk assessment. We also investigate the effect of the variants that have regulatory effects (i.e. eQTL data) on performance for risk assessment. Moreover, we compare the proposed method with the polygenic score which has been shown to be successful in different studies. Our result show that our method is significantly more powerful in risk assessment.

Our results show that multi-locus features improve prediction performance as compared to individual locus based features. We also observe that integrating functional information provided by protein-protein interaction data and expression quantitative trait loci (i.e. eQTL) data leads to more parsimonious models for risk assessment. However, inclusion of functional data does not yield significant improvement in prediction performance. This may be indicative of the limitations of genomic data in risk assessment. Furthermore, since PoCos contain loci that are related to each other in the context of a phenotype, PoCos that are discovered without the inclusion of functional information also likely contain functionally related loci. However, utilization of functional information reduces the search space to render the problem computationally feasible, and brings forward PoCos that are more functionally relevant and robust, thereby leading to more parsimonious models.

Based on the success of multi-locus genomic features in risk assessment, we conclude that combining these features with non-genetic risk factors and other biological data may lead to further improvements in risk assessment.

The proposed method is implemented in MATLAB and provided in the public domain (http://compbio.case.edu/pocos/) as open source software.

## Supporting Information

S1 Fig Effect of variance in the number of PoCos on the performance and number of selected features. (TIF)

S2 Fig Effect of p-value threshold on risk assessment performance of NetPoCos on four diseases. The x-axis shows the p-value threshold used in filtering based feature selection and the y-axis shows the area under the ROC curve (AUC) for performance in risk assessment. The curve shows the average AUC score and error bars show the standard deviation of AUC score across 5 folds in 5 different runs.
(TIF)

S3 Fig Linkage Disequilibrium (LD) distribution among selected PoCos in the prediction model. (TIF)
