# Supplementary figures and images for: PoCos: Population Covering Locus Sets for Risk Assessment in Complex Diseases
Source: PLoS Comput Biol. 2016 Nov 11;12(11):e1005195. doi: 10.1371/journal.pcbi.1005195 (PMC5105987; doi:10.1371/journal.pcbi.1005195)

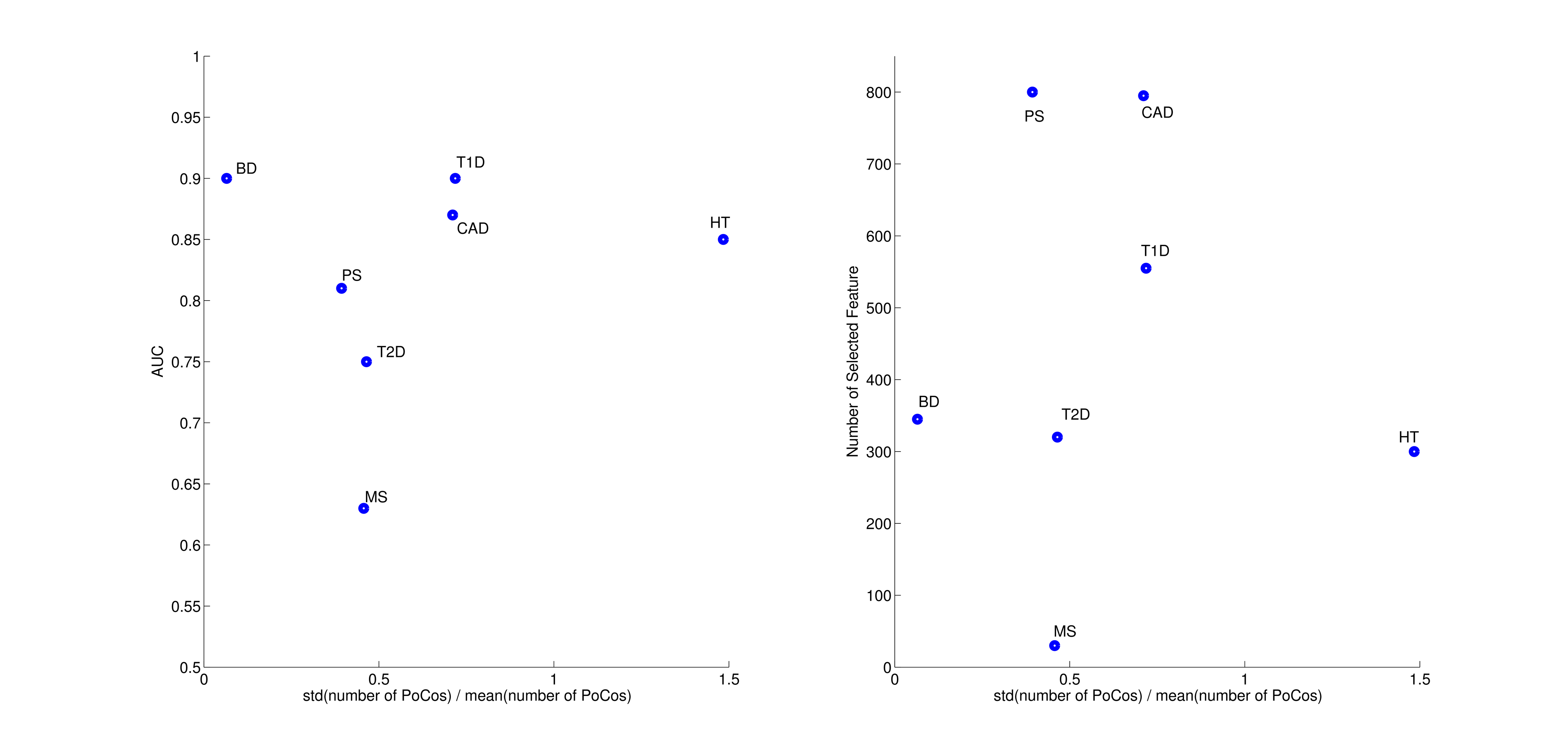

Supplement: S1 Fig — (TIF) [file pcbi.1005195.s001.tif]

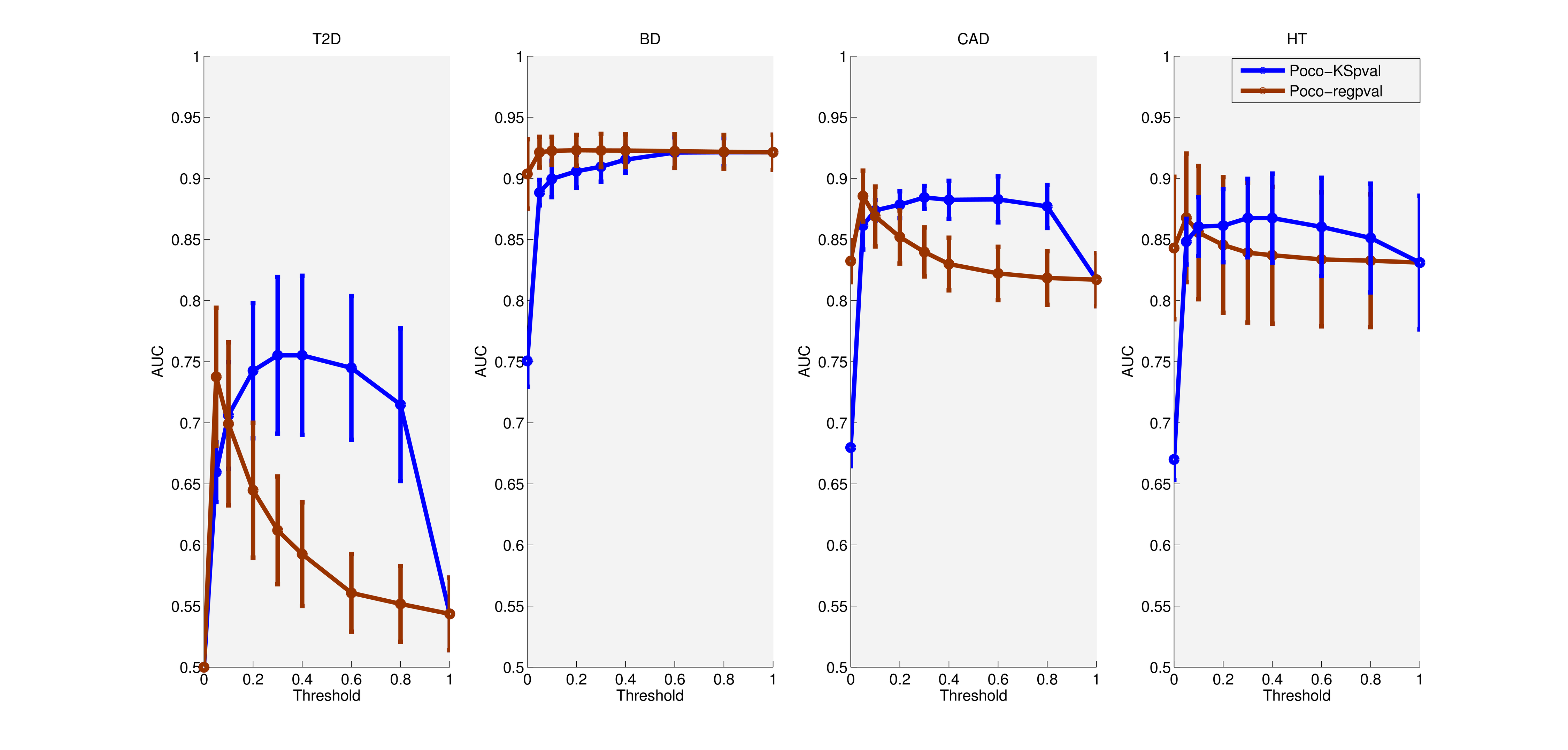

Supplement: S2 Fig — The x-axis shows the p-value threshold used in filtering based feature selection and the y-axis shows the area under the ROC curve (AUC) for performance in risk assessment. The curve shows the average AUC score and error bars show the standard deviation of AUC score across 5 folds in 5 different runs. (TIF) [file pcbi.1005195.s002.tif]

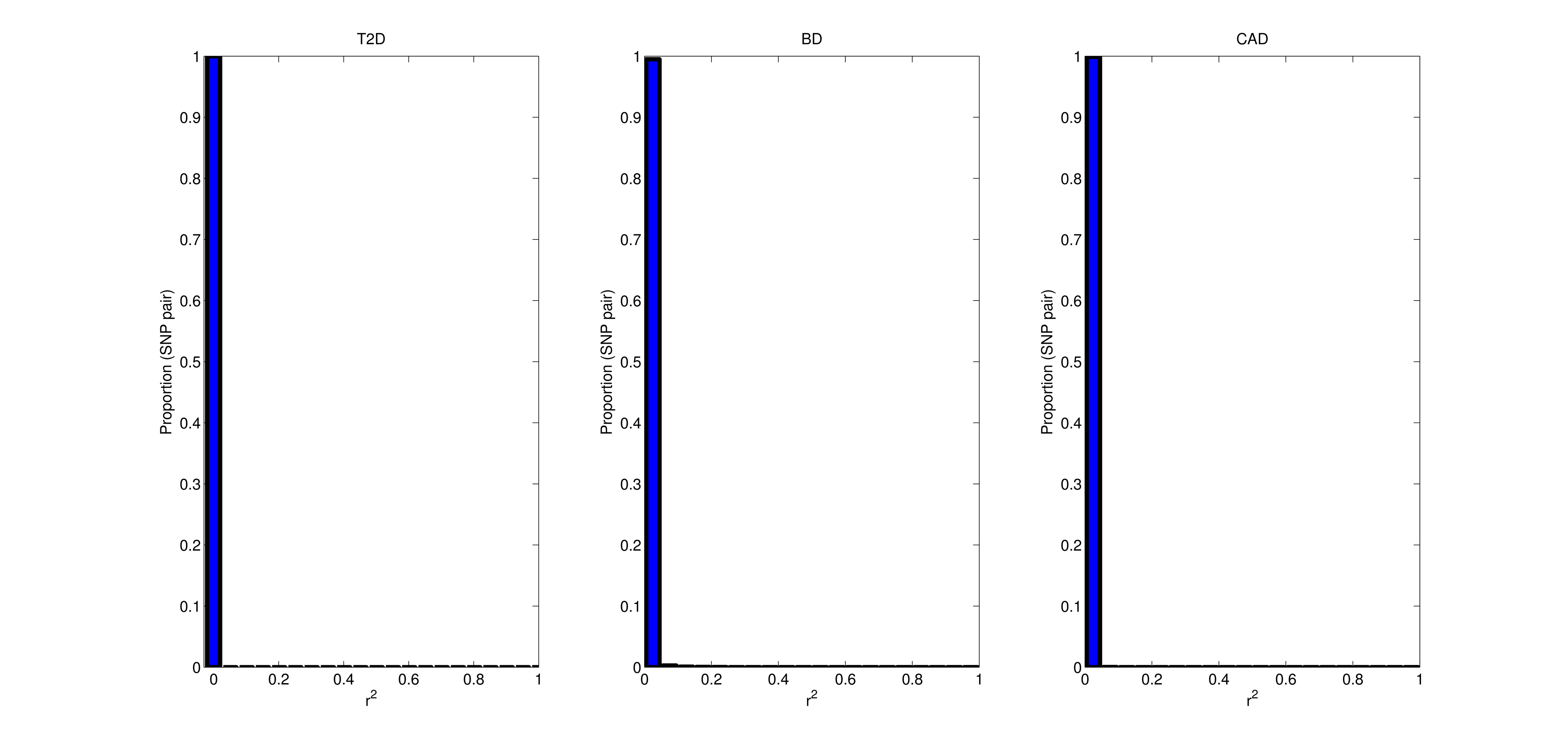

Supplement: S3 Fig — (TIF) [file pcbi.1005195.s003.tif]
